# Increased Prevalence of Blood Pressure Instability Over Twenty-Four Hours in Chronic Spinal Cord Injury

**DOI:** 10.1089/neur.2022.0007

**Published:** 2022-11-21

**Authors:** Siqi Wang, Jill M. Wecht, Beatrice Ugiliweneza, Bonnie Legg Ditterline

**Affiliations:** ^1^ Kentucky Spinal Cord Injury Research Center, University of Louisville, Louisville, Kentucky, USA.; ^2^ Department of Neurological Surgery, University of Louisville School of Medicine, University of Louisville, Louisville, Kentucky, USA.; ^3^ Department of Research and Development, James J. Peters VA Medical Center, Bronx, New York, USA.; ^4^ Department of Rehabilitation and Human Performance and Medicine, Icahn School of Medicine at Mount Sinai, New York, New York, USA.; ^5^ Department of Health Management and System Science, University of Louisville, Louisville, Kentucky, USA.

**Keywords:** autonomic dysfunction, blood pressure, blood pressure instability, cardiovascular dysregulation, spinal cord injury

## Abstract

Spinal cord injury (SCI) leads to cardiovascular dysregulation, including persistent low blood pressure (BP), orthostatic hypotension, and autonomic dysreflexia, leading to daily BP instability that may not be adequately recognized. We compared mean systolic BP, diastolic BP, and heart rate from awake and asleep measurements over a 24-h period among persons with chronic SCI (*n* = 33; 30 cervical injuries and three upper thoracic injuries), ambulatory/non-injured (Ambulatory-NI; *n* = 13), and non-injured (NI) in a wheelchair (*n* = 9). Stability of awake BP was evaluated by deviation of systolic BP from 115 mmHg and percent of systolic BP measurement within and outside of 90–140 mmHg. Variability over 24 h was compared using coefficient of variation and average real variability. Awake hyper- and hypotensive events (change in systolic BP ±20 mmHg from the median) were compared to symptoms reported by the participants corresponding to BP events. Participants with SCI had a lower percentage of awake systolic BP measurements within 90–140 mmHg than Ambulatory-NI and a greater deviation below 115 mmHg. Coefficient of variation and successive differences of awake systolic and diastolic BP were greater in SCI than Ambulatory-NI. Finally, all SCI participants had hyper- and/or hypotensive events and 88% experienced the BP events asymptomatically. In conclusion, participants with SCI had significantly greater BP instability compared with NI, with many hyper- and hypotensive events occurring without symptoms. Clinical management of BP instability, regardless of symptoms, should be a priority after SCI to reduce the risk of cardiovascular disease and improve quality of life.

## Introduction

Persons with cervical and upper thoracic spinal cord injury (SCI) can experience significantly decreased resting blood pressure (BP) compared with matched non-injured individuals (NIs). Persistent hypotension results from impaired descending sympathetic cardiovascular regulation, which leads to the inability to regulate BP in response to routine events like position changes or transfers,^1^ eating,^2^ exercising,^3^ and bowel or bladder distension.^4^ These same hypotensive persons with SCI also experience impaired circadian rhythm (i.e., absence of the nocturnal dip in BP)^5–9^ and dramatic increases in systolic BP (SBP) >20 mmHg (i.e., autonomic dysreflexia) over 24 h that can be life-threatening.^10–12^ However, the frequency and severity of BP fluctuations in the SCI population are not tracked clinically, even though these conditions are associated with cognitive decline and cerebrovascular disease in the SCI populations.^13^ Labile BP increases the risk of cardiovascular morbidity and mortality in the SCI population and is associated with increased stroke risk,^14,15^ cognitive impairments, decreased overall well-being, and increased risk of developing dementia later in life.^16^

Many, if not most, persons with SCI do not report symptoms in association with BP fluctuations during episodic hypo- or hypertensive events, and, in fact, evidence demonstrates that many persons with SCI are asymptomatic during autonomic dysreflexia.^17^ Likewise, despite reporting feeling persistently fatigued and “drained of energy,” persons with SCI do not attribute this to persistent hypotension.^18^ It is thus clinically recognized that autonomic dysreflexia and hypotension can occur asymptomatically, yet the magnitude of daily BP fluctuations are not consistently reported in persons with SCI, particularly compared to matched NI controls. Given the implications of BP instability for development of cardiovascular, cerebrovascular, and cognitive disorders and the fact that many persons with SCI experience these events asymptomatically, there is a need to quantify BP instability to improve the clinical care of persons with SCI.

Our objective was to measure BP over 24 h in participants with SCI as compared to NI controls to understand not just frequency and severity of daily hypertensive events, but also hypotensive events and to quantify BP fluctuations and instability. In addition, participants used diaries to track symptoms of BP instability and document the impact on daily life. We hypothesized that persons with SCI would experience significantly greater BP instability when compared with NIs, but that symptom reporting would not capture these fluctuations in BP.

## Methods

### Participants

This was a cross-sectional, prospective study conducted at the Kentucky Spinal Cord Injury Center (KSCIRC) and the James J. Peters Veterans Affairs Medical Center (VA). Institutional review boards at each site granted annual approval from 2008 to 2011 (VA) and from 2014 to 2021 (KSCIRC). Fifty-five participants provided informed consent: 33 participants with SCI and 22 NIs. Thirteen NIs from the KSCIRC were allowed to move freely (Ambulatory-NI); 9 NIs recruited from the VA (as part of a larger clinical trial, previously reported by Rosado-Rivera and colleagues in 2011^6^) were confined to a wheelchair while awake (Wheelchair-NI) to compare 24-h BP and heart rate (HR) without ambulatory-related changes. Inclusion criteria for all participants were: 1) >18 years of age and 2) no history of cardiopulmonary disease. Inclusion criteria for participants with SCI were: 1) chronic injury (>12 months), 2) non-ventilator dependent, 3) non-ambulatory, and 4) a drop in SBP or diastolic BP (DBP) ≥10 mmHg during a 70-degree head-up tilt. There were 30 participants with cervical (C3–C8) and 3 with high-thoracic (T2–T3) SCI classified as American Spinal Injury Association Impairment Scale (AIS) A–C.

### Data acquisition

An ambulatory BP monitor (Meditech, A & D, or Spacelabs) was programmed in advance to measure BP and HR from the brachial artery at regular intervals while awake and asleep. At the KSCIRC, BP and HR were measured every 10 or 15 min while awake and every 30 min while asleep; for participants with SCI who scheduled a bowel or bladder management program during the recording, the device was programmed to record BP and HR every 5 min during that period. At the VA, 24-h BP was recorded every 20 min while awake and every 30 min while asleep, whereas HR was monitored continuously using a Holter monitor (Brentwood IQMark Holter; Midmark Diagnostics Group, Torrance, CA). Because the raw data of the Wheelchair-NI group were reported in a previous publication, only the hourly mean will be used in the present study. Awake and asleep intervals were confirmed by participants once the recording was finished and data adjusted, if necessary. All SCI and Ambulatory-NI participants were asked to maintain a diary to document time and activities, particularly activities known to affect their BP (e.g., exercise, eating, transfers, etc.), as well as the presence of symptoms without a related event (response rate: 79% from the SCI group and 92% from the Ambulatory-NI group).

### Data analysis

Measurements were examined manually to remove artifacts before analysis with a custom program (MATLAB; The MathWorks, Inc., Natick, MA). Data were summarized into hourly means for the awake and asleep periods. Hyper- and hypotensive SBP events were calculated as an increase or decrease, respectively, of ≥20 mmHg from the median awake SBP^19,20^; BP events were compared to diary entries to track the presence/absence of symptoms.

**FIG. 1.**
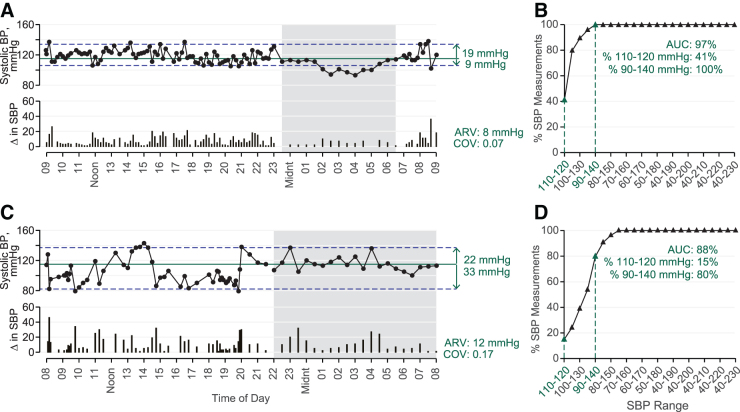
Representative 24-h blood pressure (BP) data from the Ambulatory-NI (Participant ID: N206, panels **A** and **B**) and SCI groups (Participant ID: A80, panels **C** and **D**) while awake (white background) and asleep (gray background), illustrating methods used to analyze BP stability relative to a target range (110–120 mmHg) and fluctuations between measurements. Systolic BP (SBP) measurements are represented with black circles (panels **A** and **C**, top); green line represents the middle of the target range (i.e., 115 mmHg), and dashed blue lines represent upper and lower deviation of SBP (mmHg, right). SBP measurements within and without the target range are plotted in a cumulative distribution curve (triangles, panels **B** and **C**) to quantify area under the curve (AUC), percentage of measurements within 110–120 mmHg (highlighted in green), and the percentage of measurements within 90–140 mmHg (highlighted in green). Fluctuations between successive SBP measurements (**A** and **C**, bottom) were used to calculate average real variability (ARV; right) and coefficient of variation (COV; right).

Awake SBP stability was analyzed using a validated method^21^ that quantifies variability outside the healthy awake range as determined by the lower limit of hypertension from the American Heart Association (i.e., 120 mmHg).^22^ Outcomes (Fig. 1) include deviation from the range midpoint (115 mmHg) and distribution of SBP measurements within and outside the target range (110–120 mmHg) fitted to a cumulative distribution curve. Upper and lower deviations are the absolute difference (mmHg) above and below 115 mmHg, respectively (Fig. 1A,C); if no SBP measurements are above 115 mmHg, upper deviation is 0 mmHg. To create the cumulative distribution curve, the target range was increased/decreased by 5 mmHg (e.g., from 110–120 to 105–125 mmHg), and the percentage of observations within each range relative to the entire recording was plotted.

Further analysis (Fig. 1B,D) yields area under the curve (AUC %), Y-intercept, and percentage of awake SBP measurements between 90 and 140 mmHg. The AUC reflects spread of measurements around 110–120 mmHg, and Y-intercept is the percentage within 110–120 mmHg. The 90–140 mmHg range was included for comparison because values outside that range are considered hypo- or hypertensive, respectively.^22,23^ Fluctuations in SBP, DBP, and HR while awake and asleep were quantified with coefficient of variation (COV; standard deviation [SD] divided by mean)^24,25^ and average real variability (ARV; mean of absolute successive differences).^24,26^

### Statistical analysis

Demographics were summarized (mean ± SD) and statistically compared between groups (Ambulatory-NI, Wheelchair-NI, and SCI) using analysis of variance (ANOVA) with Tukey's *post hoc* tests. Injury level and AIS were summarized with frequency (%) and compared with chi-square tests.

Systolic BP, DBP, and HR were summarized with median and quartiles. Comparisons between awake and asleep among Ambulatory-NI, Wheelchair-NI, and SCI groups were made with mixed linear models, including a random intercept per participant and random slopes for time period to account for individual variability.

Awake SBP stability measures were compared between Ambulatory-NI and SCI using linear models. Fluctuations of SBP, DBP, and HR (i.e., COV and ARV) were compared between SCI and Ambulatory-NI using mixed linear models, including a random intercept for each participant along with group, time interval, and their interaction as factors. The Wheelchair-NI group was not included in the mixed linear models for comparison because of the difference in the measurement intervals. Models were adjusted for age and body mass index (BMI). Distribution of sex was not different among the three groups and thus was not adjusted for. Estimates are presented as least square means ± standard error (SE) and, in the case of COV and ARV, compared by linear contrasts built on the interaction term.

All tests were two-sided with a significance level of 0.05. Statistical analyses were performed in SAS software (version 9.4; SAS Institute Inc, Cary, NC).

## Results

### Research participant characteristics

The Ambulatory-NI group was younger than the Wheelchair-NI and SCI groups (Table 1), and the SCI group had a lower BMI compared with the Wheelchair-NI group. Mean duration of SCI was 9.2 ± 7.8 years. Thirty-five percent of all participants were female, which did not differ significantly among the groups.

**Table 1.** Demographic and Injury Characteristics of Research Participants

			Ambulatory NI	Wheelchair NI	SCI	p value of three-group
			(*n* = 13)	(*n* = 9)	(*n* = 33)	comparison
Demographics						
	Age, years	Mean (SD)	27 (5)	41 (10)	35 (11)	0.0035
	Sex	Female, *n* (%)	5 (38)	2 (22)	12 (36)	NS
		Male, *n* (%)	8 (62)	7 (78)	21 (64)	
	Height, cm	Mean (SD)	168 (14)	172 (10)	176 (11)	NS
	Weight, kg	Mean (SD)	72 (13)	79 (16)	68 (15)	NS
	BMI	Mean (SD)	26 (2)	27 (5)	22 (5)^#^	0.0065
Injury characteristics						
	Level of injury	Cervical, *n* (%)			30 (91)	
		Thoracic, *n* (%)			3 (9)	
	AIS	A, *n* (%)			24 (73)	
		B, *n* (%)			8 (24)	
		C, n (%)			1 (3)	

^*^ *p* < 0.05 compared to Ambulatory NI; ^#^*p* < 0.05 compared to Wheelchair NI.

AIS, American Spinal Cord Association Impairment Scale; BMI, body mass index; Ambulatory NI, non-injured participants free to move; Wheelchair NI, non-injured participants in wheelchair; NS, not significant; Q1, first quartile; Q3, third quartile; SCI, spinal cord injured; SD, standard deviation.

### Aggregate blood pressure and heart rate over 24 h

**FIG. 2.**
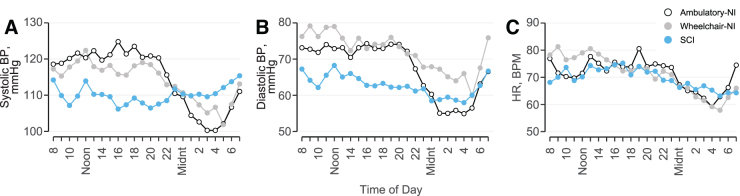
Hourly means of systolic blood pressure (BP; **A**), diastolic BP (**B**), and heart rate (HR; **C**) measured over 24 h are represented for the Ambulatory-NI (open circles), Wheelchair-NI (gray circles), and SCI (blue circles) groups. Evident is the absence of the nocturnal dip in persons with SCI compared with Ambulatory-NI and Wheelchair-NI groups. NI, non-injured; SCI, spinal cord injury.

**FIG. 3.**
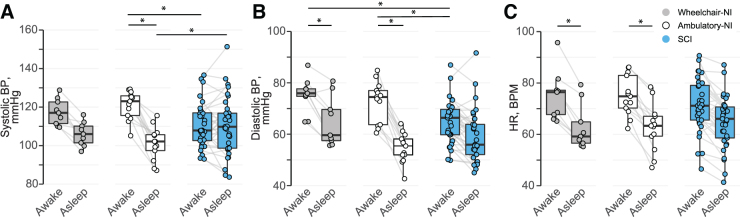
Mean systolic blood pressure (BP; **A**), diastolic BP (**B**), and heart rate (HR; **C**) while awake (left) and asleep (right) is represented for each participant in the Ambulatory-NI (open circles), Wheelchair-NI (gray circles), and SCI (blue circles) groups. Gray lines connect the awake and asleep values; box plots illustrate median and interquartile range. Systolic BP was significantly increased while awake compared with asleep in Ambulatory-NI (*p* < 0.01), but not in the Wheelchair-NI or SCI groups. Diastolic BP and HR were significantly increased while awake compared with asleep in the Ambulatory-NI (*p* < 0.001) and Wheelchair-NI (*p* < 0.05), but not the SCI group. Systolic BP while awake was significantly decreased and asleep systolic BP was significantly increased in participants with SCI (*p* < 0.05) compared with Ambulatory-NI, but not Wheelchair-NI. Diastolic BP while awake was significantly decreased in participants with SCI (*p* < 0.01) compared with both NI groups. *p < 0.05 between two groups. NI, non-injured; SCI, spinal cord injury.

Hourly means of SBP (Fig. 2A), DBP (Fig. 2B), and HR (Fig. 2C) illustrate the absence of the nocturnal dip in participants with SCI compared with Ambulatory-NI and Wheelchair-NI. SBP (Fig. 3A; Table 2) was significantly increased while awake compared with asleep in Ambulatory-NI (awake, 122 [108–133]; asleep, 103 [91–113]; *p* < 0.01), but not in the Wheelchair-NI or SCI groups. DBP (Fig. 3B) was significantly increased while awake compared with asleep in Ambulatory-NI (awake, 75 [59–82]; asleep, 56 [48–63]; *p* < 0.001) and Wheelchair-NI (awake, 77 [72–82]; asleep, 61 [53–74]; *p* < 0.05), but not the SCI group. Likewise, HR (Fig. 3C) was significantly elevated while awake compared with asleep in Ambulatory-NI (awake, 73 [63–89]; asleep, 63 [53–73]; *p* < 0.01) and Wheelchair-NI (awake, 76 [66–84]; asleep, 60 [54–66]; *p* < 0.05), but not the SCI group. Among participants with SCI, there was evidence of a decreased awake compared with asleep (i.e., reversed nocturnal dip) SBP in 15 of the 33 participants. This nocturnal reversal was evident for DBP in 7, and for HR in 8, participants with SCI and for DBP in 1 participant in the Wheelchair-NI group.

**Table 2.** Awake and Asleep Systolic Blood Pressure, Diastolic Blood Pressure, and Heart Rate

			Ambulatory NI	Wheelchair NI	SCI	p value of three-group
			(*n* = 13)	(*n* = 9)	(*n* = 33)	comparison
Systolic blood pressure, (mmHg)						
	Awake	Median [Q1–Q3]	122 [108–133]	116 [108–126]	106 [91–127]^*^	0.0036
	Asleep	Median [Q1–Q3]	103 [91–113]	104 [99–115]	110 [95–126]^*^	0.0434
	Awake vs. asleep	*p* value	0.0022	NS	NS	
Diastolic blood pressure, (mmHg)						
	Awake	Median [Q1-Q3]	75 [59–82]	77 [72–82]	63 [50–77]^^*^,#^	0.0002
	Asleep	Median [Q1-Q3]	56 [48–63]	61 [53–74]	56 [47–70]	0.032
	Awake vs. asleep	*p* value	0.0002	0.0269	NS	
Heart rate, (BPM)						
	Awake	Median [Q1–Q3]	73 [63–89]	76 [66–84]	71 [58–86]	NS
	Asleep	Median [Q1–Q3]	63 [53–73]	60 [54–66]	65 [53–77]	NS
	Awake vs. asleep	*p* value	0.0093	0.0115	NS	

^*^ *p* < 0.05 compared to Ambulatory NI; ^#^*p* < 0.05 compared to Wheelchair NI.

Ambulatory NI, non-injured participants free to move; Wheelchair NI, non-injured participants in wheelchair; NS, not significant; Q1, first quartile; Q3, third quartile; SCI, spinal cord injured; SD, standard deviation.

Average SBP (Fig. 3A; Table 2) was significantly decreased in participants with SCI while awake (*p* < 0.01) and asleep (*p* < 0.05) compared with Ambulatory-NI, but not compared with Wheelchair-NI. DBP (Fig. 3B) was significantly decreased in participants with SCI while awake (*p* < 0.01) compared with both NI groups. DBP while asleep and HR (Fig. 3C), both awake and asleep, were not significantly different among the groups.

### Symptoms of hypertensive and hypotensive events

In the SCI group, 76% (*n* = 25) of participants had both hyper- and hypotensive events according to brachial BP measurements, whereas 21% (*n* = 7) had only hypertensive events and 3% (*n* = 1) had only hypotensive events, regardless of the presence of symptoms. Of the 26 SCI participants who provided a diary, 2 reported feeling symptoms of AD alone and 1 reported feeling symptoms of AD and orthostatic hypotension, that is, 88% (23 of 26) had asymptomatic hyper- and/or hypotensive events. Comments that accompanied the asymptomatic hypertensive events as identified by BP included bowel or bladder management, exercise, position change, and pain. Comments that accompanied asymptomatic hypotensive events included position change and eating. In the Ambulatory-NI group, 31% (*n* = 4) of participants had both hyper- and hypotensive events, 23% (*n* = 3) had only hypertensive events, and 23% (*n* = 3) had only hypotensive events. None of the Ambulatory-NI participants reported symptoms of orthostatic hypotension. Comments that accompanied asymptomatic hypertensive events included daily physical activities, exercise, and caffeine pills.

### 24-h systolic blood pressure stability

**FIG. 4.**
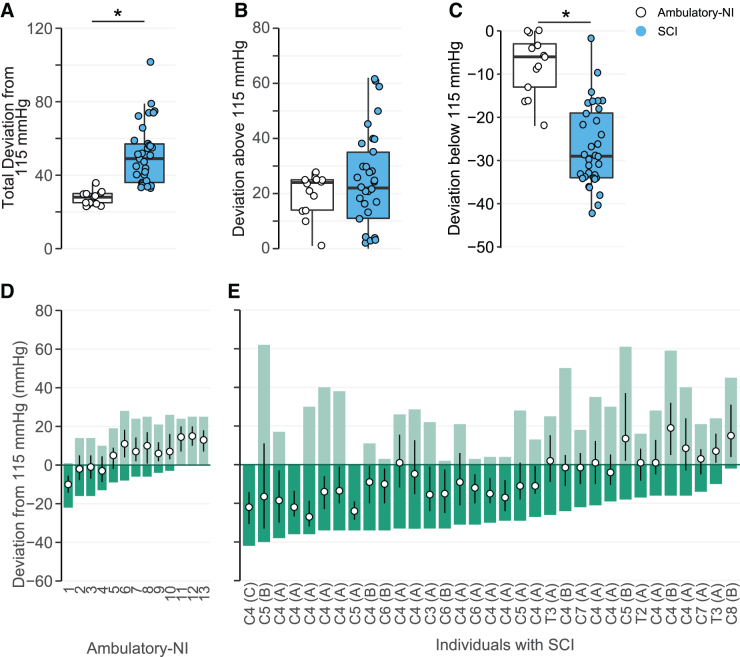
Mean systolic blood pressure (SBP) total deviation around (**A**), deviation above (**B**), and deviation below (**C**) the middle of the target range (115 mmHg) while awake is represented for participants in the Ambulatory-NI (open circles) and SCI (blue circles) groups; box plots illustrate median and interquartile range. Bars illustrate deviation above (light green) and below (dark green) 115 mmHg for each participant in the Ambulatory-NI (**D**) and SCI (**E**) groups with median (open circle) and interquartile range. Total deviation (*p* < 0.01) and deviation below 115 mmHg (*p* < 0.001) were significantly greater in participants with SCI compared with Ambulatory-NI. Additionally, whereas the total deviation of SBP was evenly distributed around the median in Ambulatory-NI (D), this was not the case in most participants with SCI (E), where the median SBP was skewed toward the lower deviation despite a homogenous group of injuries. *p < 0.05 between two groups. NI, non-injured; SCI, spinal cord injury.

Awake SBP stability relative to a normative range was decreased in participants with SCI compared with Ambulatory-NI. Total deviation (Fig. 4A; Supplementary Table S1) was significantly increased in participants with SCI (50 ± 3 mmHg; *p* < 0.01) compared with Ambulatory-NI (33 ± 5 mmHg). Although deviation above 115 mmHg (Fig. 4B) did not differ significantly between groups, deviation below 115 mmHg (Fig. 4C) was significantly increased in the SCI group (−26 ± 1 mmHg) compared to the Ambulatory-NI (−13 ± 2 mmHg; *p* < 0.001). Additionally, whereas the total deviation of SBP was evenly distributed around the median in the 13 Ambulatory-NI participants (Fig. 4D), this was not the case in most participants with SCI (Fig. 4E); in fact, median SBP was skewed toward the lower deviation in 20 of the 33 participants, despite a homogenous group of injuries.

**FIG. 5.**
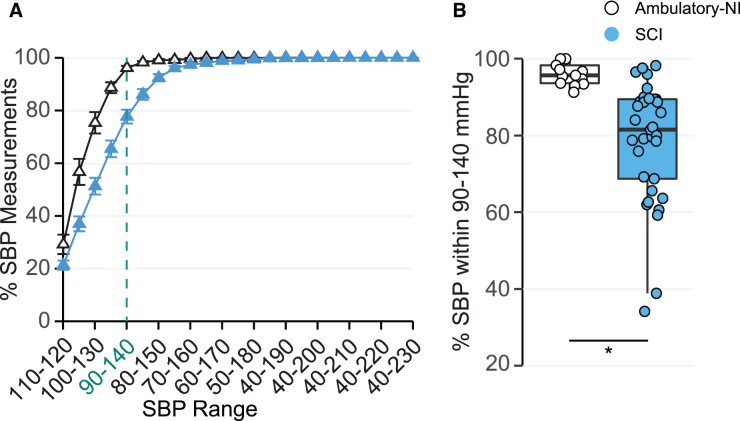
Mean ± SE awake systolic blood pressure (SBP) measurements within and outside the target range are plotted in a cumulative distribution curve (**A**) for the Ambulatory-NI (open triangles) and SCI (blue triangles) groups. Green dashed line indicates the SBP range 90–140 mmHg. Percentage of SBP measurements within 90–140 mmHg (**B**) while awake is represented for the Ambulatory-NI (open circles) and SCI (blue circles) groups; box plots illustrate median and interquartile range. Awake AUC (*p* < 0.05) and percentage of SBP measurements within 90–140 mmHg (*p* < 0.05) were significantly decreased in participants with SCI compared with the Ambulatory-NI group. *p < 0.05 between two groups. NI, non-injured; SCI, spinal cord injury; SE, standard error.

Awake AUC (Fig. 5A; Supplementary Table S1) was significantly decreased in participants with SCI (90.0 ± 0.7%; *p* < 0.05) when compared with the Ambulatory-NI group (93 ± 1%). The percentage of awake SBP measurements that fell between 90 and 140 mmHg (Fig. 5B) was significantly lower in the SCI group (80 ± 2%) compared with Ambulatory-NI (91 ± 4%; *p* < 0.05), whereas the Y-intercept was not significantly different between groups (Fig. 5A; Supplementary Table S1).

### Blood pressure and heart rate fluctuations

Fluctuations in SBP were significantly increased in participants with SCI compared with Ambulatory-NI. While awake, ARV (Supplementary Fig. S1A; Table 3) was significantly increased in the SCI group compared to Ambulatory-NI. In addition, the COV of SBP (Table 3) was significantly increased in participants with SCI compared with Ambulatory-NI. Group differences for asleep SBP COV and ARV were not significant. Similar findings were evident for diastolic ARV and COV both awake and asleep, whereas neither ARV nor COV for HR were significantly different during awake or asleep periods when comparing the SCI to Ambulatory-NI group (Table 3).

**Table 3.** Fluctuations of Blood Pressure and Heart Rate

			Ambulatory NI	SCI	p value of group comparison
			(*n* = 13)	(*n* = 33)	
Systolic blood pressure					
	ARV (mmHg)	Awake	9.80 ± 1.25	12.90 ± 0.69	0.0375
		Asleep	8.80 ± 1.25	10.10 ± 0.69	NS
	COV (AU)	Awake	0.0976 ± 0.0120	0.1462 ± 0.0066	0.0013
		Asleep	0.0933 ± 0.0120	0.0939 ± 0.0066	NS
Diastolic blood pressure					
	ARV (mmHg)	Awake	7.40 ± 0.78	9.30 ± 0.43	0.0423
		Asleep	5.60 ± 0.78	7.40 ± 0.43	NS
	COV (AU)	Awake	0.1143 ± 0.0134	0.1860 ± 0.0074	<0.0001
		Asleep	0.1079 ± 0.0134	0.1473 ± 0.0074	0.0168
Heart rate					
	ARV (mmHg)	Awake	8.30 ± 0.83	7.20 ± 0.45	NS
		Asleep	5.80 ± 0.83	5.20 ± 0.45	NS
	COV (AU)	Awake	0.1569 ± 0.0169	0.1543 ± 0.0092	NS
		Asleep	0.1188 ± 0.0169	0.1041 ± 0.0092	NS

Data are presented as estimate mean ± SE.

Ambulatory NI, non-injured participants free to move; ARV, average real variability, that is, mean of absolute successive differences between measurements; AU, arbitrary unit; COV, coefficient of variation; NS, not significant; SCI, spinal cord injured; SE, standard error.

## Discussion

Persons with cervical and high-thoracic SCI experience significantly greater BP instability than NIs, as evident in the significantly increased BP deviation, range, and fluctuations between absolute successive measurements. More important, much of this fluctuation between hypertension/hypotension is asymptomatic: Many report having adjusted to the daily BP dysregulation. Labile BP increases the risk of stroke and development of cardiovascular disease and has strong implications for future development of cognitive diseases like dementia^14–16^; thus, clinical management of BP instability regardless of symptoms should be a priority.

### Blood pressure instability in spinal cord injury

Hourly means of SBP, DBP, and HR confirm the absence of the nocturnal dip in BP and HR in participants with SCI compared with the Ambulatory-NI and Wheelchair-NI groups. This finding most likely reflects altered circadian BP variability from sympathetic nervous system impairment. Interestingly, awake SBP was significantly increased compared with asleep in Ambulatory-NI, but not in the Wheelchair-NI or SCI groups. Likewise, awake SBP was significantly decreased in participants with SCI compared with Ambulatory-NI, whereas awake DBP was significantly decreased in participants with SCI compared with both Ambulatory-NI and Wheelchair-NI. This is likely attributable to the Wheelchair-NI group limiting their movement, which minimizes changes in SBP because of physical activity and position changes. DBP, however, is less influenced by movement and more related to peripheral vascular resistance.^27^

Aside from the circadian impairment exhibited by persons with SCI, the mean awake and asleep BP and HR results reported herein would fall well within diagnostically “healthy” values.^28^ This is because the consensus statement on 24-h BP is prognostic for cardiovascular disease risk secondary to hypertension in NIs. Although it is known that persons with SCI do not exhibit persistent hypertension, our data demonstrate that they do not exhibit persistent hypotension either: They experience labile BP that varies between hyper- and hypotension multiple times a day. Therefore, averaging BP and HR over 24 h does not capture the degree of cardiovascular instability persons with SCI experience; as such, closer evaluation is necessary.

When quantifying 24-h BP stability, our data make evident that persons with SCI exhibit significantly greater instability compared with NIs: The range of BP measurements and variability between measurements are significantly greater. Total deviation and deviation below 115 mmHg were significantly increased in participants with SCI, most likely reflecting persistent hypotension interspersed with bouts of hypertension. Even though deviation above 115 mmHg was not significantly different, the median and interquartile ranges illustrate dramatically different presentations: The upper deviation contains the median and interquartile range for most Ambulatory-NI, indicating that the upper deviation is related to oscillation in SBP around the median. However, the median and interquartile range are contained within the lower deviation and is independent of upper deviation in the SCI group. Our data indicate that persons with SCI can experience dramatic increases in BP unrelated to their resting BP, whereas Ambulatory-NI deviation is dependent on resting BP.

Whereas awake AUC was significantly decreased in participants with SCI when compared with Ambulatory-NI, the percentage of SBP measurements within 110–120 mmHg did not differ significantly, which reflects the wider range of awake SBP in the SCI group. Only 27% of awake NI SBP measurements fell within 110–120 mmHg, but 91% were within 90–140 mmHg. By contrast, 22% of awake SBP measurements were within the 110–120 mmHg range, but only 80% fell between 90 and 140 mmHg in the SCI group. A devastating finding from the present data set was that, to capture all SCI SBP observations, the range must be expanded to 60–220 mm Hg.

In addition to significantly greater daily BP instability, persons with SCI also experience significantly more fluctuations. Increased COV (the ratio of SD to the mean) in persons with SCI reflects an increased dispersion of SBP measurements around the mean compared with Ambulatory-NI. Likewise, ARV was significantly increased in participants with SCI when compared with Ambulatory-NI, indicating that variability between successive measurements was greater despite less movement.

### Impact of blood pressure instability on daily life

Despite the significantly greater BP instability and fluctuations experienced by persons with SCI, 88% of SCI participants had hyper- and hypotensive events asymptomatically, similar to previous research.^2,18^ This demonstrates that BP instability is significantly worse in persons with SCI, but goes unnoticed^29^ until events are severe enough to require medical intervention.^30,31^ The chronic and severe instability that occurs daily is thus largely untreated,^32,33^ despite the increased cardiovascular morbidity and mortality in persons with SCI. This is clinically significant because excessive fluctuations in SBP can be more damaging to organs than persistent hypertension. Longitudinal studies have found the difference between successive SBP measurements to be equally predictive of stroke as the highest SBP measurement,^34^ and that persons who experience stroke or ischemic events were more likely to have been previously diagnosed with “episodic” hypertension rather than “stable” hypertension.

Additionally, the risk of stroke^35^ and cognitive decline^36,37^ is more significantly correlated to SBP variability and arterial stiffness than elevated mean SBP. This is because excessive fluctuations in SBP are accompanied by vascular stiffness,^38,39^ a condition accelerated by age in which elastic connective tissue has degraded.^40,41^ The ability of the arteries to absorb BP and recoil with each heartbeat is diminished, thus increasing the risk of rupture.^42^ Persons with SCI have increased arterial stiffness^43^ along with increased risk of dementia and stroke, which makes the asymptomatic BP instability and fluctuations we report herein of clinical importance.

### Future direction and limitations

Clinical care of persons with SCI should involve therapeutic approaches to maintain BP within a normative range to mitigate functional consequences of chronic hypotension as well as acutely treat autonomic dysreflexia. As new therapeutic interventions are tested for their ability to modulate BP, effects on stability must be evaluated to understand implications for long-term cardiovascular disease risk. Additionally, the prognostic value of significantly increased DBP fluctuations reported herein are unknown. Elevated DBP variability in relation to cardiovascular mortality in NIs^44–46^ is unclear, and, as such, clinical significance as it relates to persons with SCI, particularly when asleep, needs to be studied further. In the present study, a limitation is that there is a large difference in the number of participants between SCI and NI groups. Although for all the statistical tests and models used, equal or even sample sizes are not an assumption or a requirement for validity, a matched number of participants could yield a larger power generally.

Finally, nearly all the persons with SCI who participated had cervical AIS A or B SCI, and they had demonstrated BP dysregulation during the head-up tilt test; comparisons with low-level and ambulatory SCI, and with those who do not have BP dysregulation during the head-up tilt test, should be investigated to better understand the relationship between BP instability, degree of motor impairment, and autonomic cardiovascular dysregulation, as well as other conditions that affect cardiovascular mortality (e.g., dyslipidemia, atherosclerosis, etc.).

## Supplementary Material

Supplemental data

Supplemental data

## Abbreviations Used

AIS: American Spinal Cord Association Impairment Scale
ANOVA: analysis of variance
ARV: average real variability
AUC: area under the curve
BMI: body mass index
BP: blood pressure
COV: coefficient of variation
DBP: diastolic blood pressure
HR: heart rate
KSCIRC: Kentucky Spinal Cord Injury Center
NIs: non-injured individuals
SBP: systolic blood pressure
SCI: spinal cord injury
VA: James J. Peters Veterans Affairs Medical Center
