# Supplementary material for: Increased Prevalence of Blood Pressure Instability Over Twenty-Four Hours in Chronic Spinal Cord Injury
Source: Neurotrauma Rep. 2022 Nov 21;3(1):522–33. doi: 10.1089/neur.2022.0007 (PMC9718427; doi:10.1089/neur.2022.0007)
Supplement: Supplemental data [file Supp_TableS1.docx]

| Supplemental Table 1. Systolic blood pressure stability while awake | | |  |
| --- | --- | --- | --- |
|  | *Ambulatory NI* | *SCI* | *p-value of group comparison* |
|  | *(n = 13)* | *(n = 33)* |  |
| Total deviation from 115 mmHg, (mmHg) | 32.5 ± 4.88 | 49.5 ± 2.61 | 0.0058 |
| Deviation above 115 mmHg, (mmHg) | 19.8 ± 5.46 | 23.9 ± 2.92 | NS |
| Deviation below 115 mmHg, (mmHg) | 12.7 ± 2.47 | 25.6 ± 1.32 | <.0001 |
| AUC, (%) | 93.3 ± 1.28 | 89.6 ± 0.69 | 0.0218 |
| Y-intercept, (%) | 27.1 ± 3.62 | 22.5 ± 1.94 | NS |
| Percent within 90-140 mmHg, (%) | 91.4 ± 4.41 | 79.9 ± 2.36 | 0.0356 |
| Data are presented as estimate mean ± SE. | | | |
| Ambulatory NI: non-injured participants free to move; AUC: area under the curve; NS: not significant; SCI: spinal cord injured; SE: standard error. | | | |
