# Supplementary material for: Increased Prevalence of Blood Pressure Instability Over Twenty-Four Hours in Chronic Spinal Cord Injury
Source: Neurotrauma Rep. 2022 Nov 21;3(1):522–33. doi: 10.1089/neur.2022.0007 (PMC9718427; doi:10.1089/neur.2022.0007)
Supplement: Supplemental data [file Supp_FigS1.docx]

**Supplemental Figure 1**. Mean average real variability (ARV) of awake systolic blood pressure (BP, **A**) and diastolic BP (**B**) is represented for the Ambulatory-NI (open circles) and SCI (blue circles) groups; boxplots illustrate median and interquartile range. Awake, ARV of systolic BP (p < .05) and diastolic BP (p < .05) were significantly greater in the SCI group compared with Ambulatory-NI. *: P<.05 between two groups.
